# Pullout Strength of Pedicle Screws Inserted Using Three Different Techniques: A Biomechanical Study on Polyurethane Foam Block

**DOI:** 10.3390/bioengineering10060660

**Published:** 2023-05-30

**Authors:** Lien-Chen Wu, Yueh-Ying Hsieh, Fon-Yih Tsuang, Yi-Jie Kuo, Chia-Hsien Chen, Chang-Jung Chiang

**Affiliations:** 1 Department of Orthopaedics, Shuang Ho Hospital, Taipei Medical University, New Taipei City 23561, Taiwan; d98548019@tmu.edu.tw (L.-C.W.); 11154@s.tmu.edu.tw (Y.-Y.H.); chiaxian@tmu.edu.tw (C.-H.C.); 2 Department of Orthopaedics, School of Medicine, College of Medicine, Taipei Medical University, Taipei City 11031, Taiwan; benkuo5@tmu.edu.tw; 3 Graduate Institute of Biomedical Materials and Tissue Engineering, College of Biomedical Engineering, Taipei Medical University, Taipei City 11031, Taiwan; 4 Division of Neurosurgery, Department of Surgery, National Taiwan University Hospital, Taipei City 100225, Taiwan; tsuangfy@ntu.edu.tw; 5 Spine Tumor Center, National Taiwan University Hospital, Taipei City 100225, Taiwan; 6 Department of Orthopedic Surgery, Wan Fang Hospital, Taipei Medical University, Taipei City 11696, Taiwan; 7 School of Biomedical Engineering, College of Biomedical Engineering, Taipei Medical University, Taipei City 11031, Taiwan

**Keywords:** pedicle screw, insertion depth adjustment, pullout strength, biomechanical study

## Abstract

Pullout strength is an important indicator of the performance and longevity of pedicle screws and can be heavily influenced by the screw design, the insertion technique and the quality of surrounding bone. The purpose of this study was to investigate the pullout strength of three different pedicle screws inserted using three different strategies and with two different loading conditions. Three pedicle screws with different thread designs (single-lead-thread (SLT) screw, dual-lead-thread (DLT) screw and mixed-single-lead-thread (MSLT) screw) were inserted into a pre-drilled rigid polyurethane foam block using three strategies: (A) screw inserted to a depth of 33.5 mm; (B) screw inserted to a depth of 33.5 mm and then reversed by 3.5 mm to simulate an adjustment of the tulip height of the pedicle screw and (C) screw inserted to a depth of 30 mm. After insertion, each screw type was set up with and without a cyclic load being applied to the screw head prior to the pullout test. To ensure that the normality assumption is met, we applied the Shapiro–Wilk test to all datasets before conducting the non-parametric statistical test (Kruskal–Wallis test combined with pairwise Mann–Whitney-U tests). All screw types inserted using strategy A had a significantly greater pullout strength than those inserted using strategies B and C, regardless of if the screw was pre-loaded with a cyclic load prior to testing. Without the use of the cyclic pre-load, the MSLT screw had a greater pullout strength than the SLT and DLT screws for all three insertion strategies. However, the fixation strength of all screws was reduced when pre-loaded before testing, with the MSLT screw inserted using strategy B producing a significantly lower pullout strength than all other groups (*p* < 0.05). In contrast, the MSLT screw using insertion strategies A and C had a greater pullout strength than the SLT and DLT screws both with and without pre-loading. In conclusion, the MSLT pedicle screw exhibited the greatest pullout strength of the screws tested under all insertion strategies and loading conditions, except for insertion strategy B with a cyclic pre-load. While all screw types showed a reduced pullout strength when using insertion strategy B (screw-out depth adjustment), the MSLT screw had the largest reduction in pullout strength when using a pre-load before testing. Based on these findings, during the initial screw insertion, it is recommended to not fully insert the screw thread into the bone and to leave a retention length for depth adjustment to avoid the need for screw-out adjustment, as with insertion strategy B.

## 1. Introduction

Screw loosening is a common complication associated with pedicle screws used for spinal fixation, particularly in osteoporotic patients [1,2,3]. Early screw loosening may lead to fixation failure and require revision surgery to correct. Recent developments in biomaterials and processing methods have improved the structural stiffness of pedicle screws and allowed for the mechanical properties to be adjusted for different applications [4,5]. Increasing the rigidity of the screw/rod construct can offer immediately stability to the fixed segments and reduce the risk of fusion failure [6] but may also alter the stress distribution to surrounding bone. A stiff pedicle screw system will bear much of the load placed on the implanted segment and reduce the load on the bone, which can increase the force at the bone–screw interface. This increased force may cause the collapse of the bone structure or thread damage (since the loading was transferred through the bone–screw interface), potentially leading to pedicle screw loosening [7]. Numerous designs for the screw thread have been developed to try to improve the screw purchase [8,9], with the most common commercial screw threads being single-lead-thread (SLT), dual-lead-thread (DLT), mixed-single-lead-thread (MSLT) and proximal-unthread-dual-thread (PUDL).

The design of pedicle screws, the quality of surrounding bone and the surgical insertion technique are the main parameters affecting the screw holding strength [9,10]. Pedicle screw fixation is used to treat various spinal diseases, and the depth of screw insertion is usually adjusted intraoperatively to allow the rod to connect to the screw head and conform to the deformity of the spinal segments. A biomechanical study by Ying [11] reported that adjusting the height of the screw after cement augmentation led to a reduction in pullout strength. Similarly, using in vitro and mechanical testing, Matityahu et al. [12] found that repetitive insertion of cortical screws in the same hole reduced the pullout strength of the screw, with more insertions corresponding to a sequential loss in holding strength. Thus, altering the depth or purchase of the original screw after insertion can have a demonstrable negative effect on the pullout strength. Pullout strength test using synthetic bone is a common parameter for assessing the stability of pedicle screws during preclinical testing [9,13]. However, to date, no studies have compared the pullout strength of pedicle screws with different thread designs when the screw height is adjusted after implantation.

Synthetic bones are commonly used in the evaluation of implant safety and function; however, they may not accurately reflect the physical loading conditions. Nevertheless, pullout strength per ASTM standard can be used as an indicator of the holding capacity of a screw in a material during preclinical testing.

The purpose of this study was to investigate changes in the pullout strength of pedicle screws with different thread designs after adjusting the insertion depth. The hypothesis was that the pullout strength would decrease after partial withdrawal of the screw, particularly for complex thread designs.

## 2. Materials and Methods

**Figure 1:**
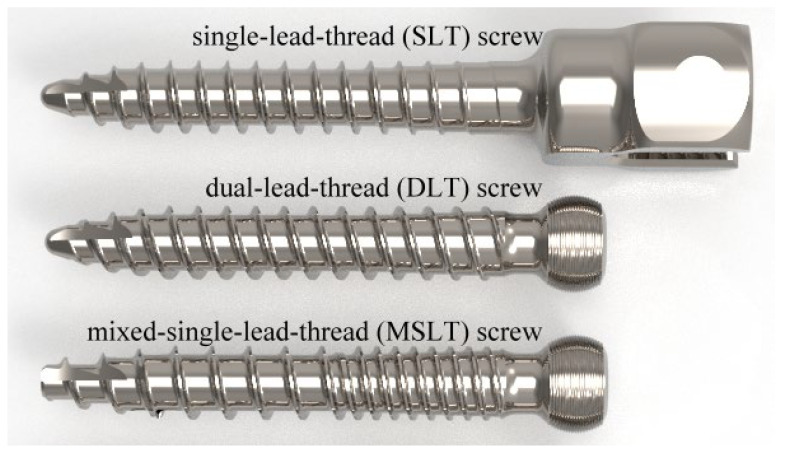
Three different commercial pedicle screws assessed in this study (OCTOPODA, Bricon GmbH).

Rigid polyurethane foam blocks (40 mm × 40 mm × 40 mm, Grade 20 (0.32 g/cm^3^) (1522-03; Sawbones, Pacific Research Laboratories Inc., Vashon, WA, USA) were used for all tests in this study [14]. A density of 0.32 g/cm^3^ was chosen from the literature [15,16] demonstrating comparable compressive mechanical characteristics and screw pullout strength with non-osteoporotic cancellous vertebral bone. The type and density of the synthetic bone conform to ASTM F1839-08 [17]. An axial pullout test was conducted to compare three distinct types of commercial pedicle screws (OCTOPODA, Bricon GmbH, Wurmlingen, Germany, Figure 1), namely a single lead thread (SLT) screw (monoaxial pedicle screw), dual lead thread (DLT) screw (polyaxial pedicle screw) and mixed single lead thread (MSLT) screw (polyaxial pedicle screw). Table 1 details the thread features for each screw, which all had a shaft length of 35 mm and diameter of 6.0 mm and were composed of a titanium alloy (Ti6Al4V ELI). While the thread on the SLT screw is arranged in a single helical pattern along the entire length of the screw, the DLT screw has a double-helical thread. With the thread arranged in a single helical pattern along the proximal core of the MSLT screw, the proximal portion has a narrower pitch than the distal core.

**Figure 2:**
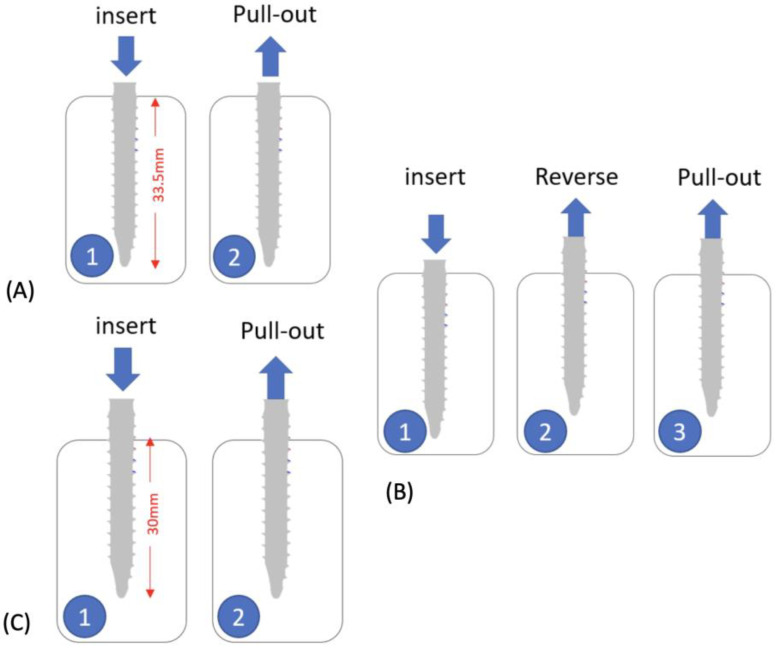
Pedicle screws inserted using three different strategies. (**A**) Insert the screw to a depth of 33.5mm, then pull out. (**B**) Insert the screw to a depth of 33.5 mm, reverse the screw by 3.5 mm, then pull out. And (**C**) Insert the screw to a depth of 30 mm, then pull out.

Pullout testing was performed according to ASTM F543 [18] using a custom-made pulling jig and an MTS axial/torsional load cell (model 662.20H-05) attached to an MTS MiniBionix testing system (MTS Systems Corporation, Eden Prairie, MN, USA). The machine was capable of generating an axial force up to 25 kN and a torque of 250 Nm. Using a 4.2 mm diameter drill bit, a single hole was pre-drilled in the center of each foam block perpendicular to the surface and along the longitudinal axis of the pedicle screw. The pedicle screws were inserted using three different strategies (Figure 2) at a rate of 3 rev/min. Strategy A is to insert the screw to a depth of 33.5 mm; strategy B is to insert the screw to a depth of 33.5 mm and then reverse the screw by 3.5 mm to simulate a surgeon adjusting the tulip height of the pedicle screw, and strategy C is to insert the screw to a depth of 30 mm.

**Figure 3:**
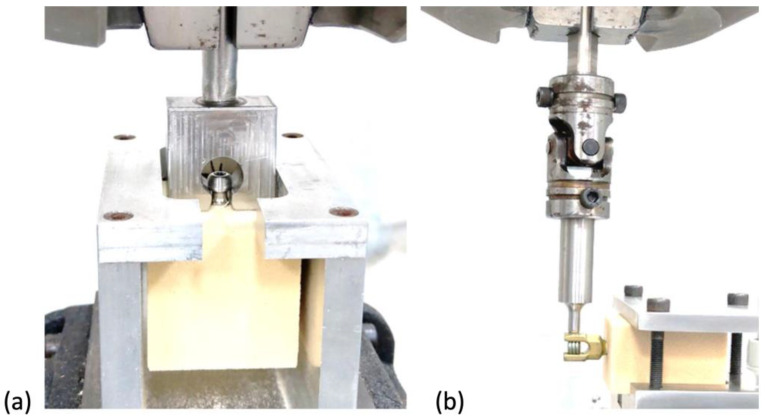
(**a**) Setup for pull-test; (**b**) Application of caudal-cephalad displacement on the screw head.

Pullout testing was performed on six groups of samples, where groups 1 to 3 (screw type SLT, DLT and MSLT, respectively) were tested after screw insertion, and groups 4 to 6 (screw type of SLT, DLT and MSLT, respectively) were first subjected to a ±5 mm caudal-cephalad displacement on the screw head, followed by a cyclic load for 5000 cycles at 3 Hz [19] after screw insertion prior to the pullout test. Using the setup in Figure 3a, each screw was pulled away from the test block at a loading rate of 1 mm/s. The maximum load recorded prior to failure by any means was considered to be the fixation strength of each screw. Five screws were tested with each group and insertion strategy, using a different sawbone sample and screw for each test.

To ensure that the dataset in each group follows a normal distribution (*p* > 0.05), we checked them using the Shapiro–Wilk test before performing a non-parametric statistical test. The non-parametric statistical evaluation (Kruskal–Wallis test combined with pairwise Mann–Whitney-U tests) was used to determine significant differences between individual means with a significance level of 0.05.

## 3. Results

**Figure 4:**
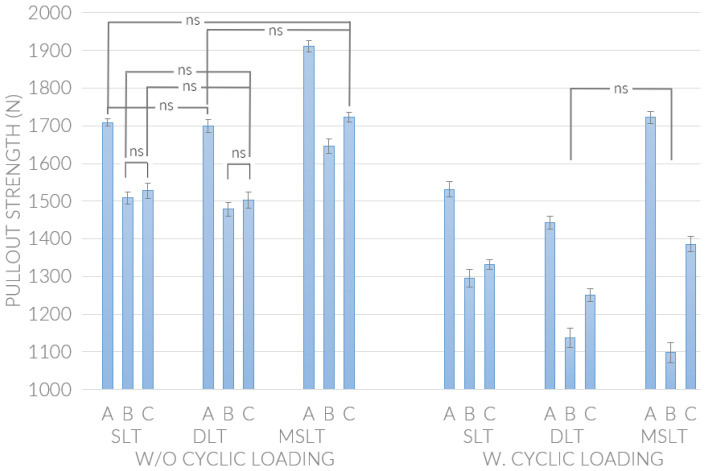
Pullout strength of screws inserted using three different insertion strategies. Results where no significant (ns) difference was found within each group are identified.

For the SLT screw group without cyclic loading, the average pullout strength of screws inserted using strategy A was significantly (*p* < 0.05) greater than those inserted using strategies B and C (Table 2, Figure 4), while the difference between strategies B and C was not significant (*p* > 0.05). Inserting the DLT screws using strategy B without cyclic loading resulted in a significantly lower pullout strength (*p* < 0.05) than strategy A. Using screw insertion strategy B in the MSLT screw group without cyclic loading did not results in a higher pullout strength than strategy A and C.

Applying a cyclic load to the screw after insertion (groups 4 to 6) using strategy B resulted in a significantly lower pullout strength (*p* < 0.05) than the other insertion strategies for all screw types (Table 3 and Figure 4). However, among the screws inserted using strategy B, the SLT screw had a significantly greater pullout strength than the other designs (*p* < 0.05). For strategies A and C without or with cyclic loading, the MSLT screw had a greater pullout strength than both SLT and DLT screws.

## 4. Discussion

The depth of screw insertion is typically adjusted during the surgery to enable the connection of the rod to the screw head and to accommodate the deformity of the spinal segments. Compared to strategy C, strategy B resulted in a greater decrease in pullout strength for all screw types without cyclic loading, with the DLT screw showing the greatest reduction. Pullout strength is an important factor affecting the screw holding captivity and is typically evaluated using ASTM F543 [9,10,11,12,13]. The results showed that the MSLT screw had greatest pullout strength among the three screw types for all insertion methods, while the difference between the SLT and DLT groups was not significant when using the same insertion method. The design of the MSLT screw allows for more contact at the bone–screw interface, which is a primary factor for increasing the pullout strength [9].

Adjusting the depth of the screw after insertion was found to reduce the pullout strength of all screw types. The greatest reduction in pullout strength was found in the MSLT group after screw-out depth adjustment. In the SLT and DLT groups, the average pullout strengths in depth adjustment of screw-out were lower than the values in the located screw depth by single screw-in, but no significant difference of pullout strength were found by using different strategies of screw depth insertion. This is likely because the insertion path was tapped by the first insertion and reversing the screw, particularly with the mixed-thread screw (MSLT), caused further damage to the bone interface. Wadhwa et al. [20] reported a similar finding where removing a screw and inserting a new one in the same hole reduced the pull out strength and also altered the biomechanical movements (axial rotation and flexion/extension) around the implanted region. This can lead to a lower fixation stability, increasing the risk of screw loosening and fixation failure.

**Figure 5:**
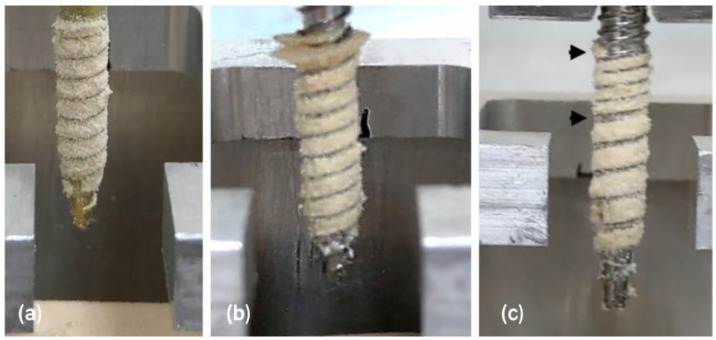
Comparison of material debris embedded in screw threads (**a**) SLT, (**b**) DLT and (**c**) MSLT screws after the application of cyclic loading prior to pullout testing. The black arrows point to the gaps in the bone surrounding the screw thread.

Applying a cyclic load to the screw before the pullout test caused a significant drop in pullout strength across all tested groups, which indicated that the screw–bone interface is affected by cyclic loading. As with the screws that were not pre-loaded, reversing the screw (strategy B) prior to the pullout test reduced the screw–bone holding capacity, with the MSLT group having the lowest pullout strength among the three groups. The considerable reduction in pullout strength between strategies A and B in the MSLT group is likely due to the part proximal path of hole being tapped twice (at screw-in and screw-out) and the cyclic load damaging the material at the screw–bone interface. As shown in Figure 5, the cyclic loading on the MSLT screw caused greater damaged to the insertion hole than the SLT and LDT screws, which is evident by the gaps in the bone withdrawn around the screw thread (black arrows). Karami et al. [21] indicated that cyclic loading is an important step for evaluating the pullout strength of screws because it represents loading on the screw head that may occur during a patient’s daily activities.

This study has some limitations. First, a synthetic bone block was used instead of cadaveric bone for the pullout testing. While synthetic bone may not be truly representative of real bone, the foam models used for pullout strength only calculated the contribution of the vertebral body and did not represent the cortical-cancellous region of the pedicle, nor did they consider the non-uniform bone density and morphology of the pedicle in real conditions; however, it does have some advantages, such as negligible inter-specimen variability, low cost, ready availability and minimal specimen preparation, which complement the aim of this study to compare the pullout strength of different pedicle screws. Two important limitations of our study were that the sample size was small (*n* = 5 in each group) and the morphology of the pedicle bone and the cortical shell of the vertebral body was not considered, and morphology is an important factor in influencing the loading pattern physiologically. In our study, pullout strength per ASTM standard was considered the indicator to represent the initial holding capacity of pedicle screws. Other real physical simulation loading conditions, such as daily cycle loadings, were not considered. Moreover, it is important to note that this study only evaluated three thread designs of a specific commercially available pedicle screw series.

## 5. Conclusions

Without applying a cyclic load to the screw head prior to pullout testing, the MSLT screw had the greatest pullout strength of all screws inserted using strategy A. However, applying a cyclic load before the pullout test caused a drastic reduction in the holding capacity, and the MSLT screw inserted using strategy B had the lowest pullout strength of all screws regardless of insertion method. In addition, the strategy B insertion method was found to produce a lower pullout strength than the other insertion methods both with and without cyclic loading. Based on these findings, we recommend not driving the full screw thread into the pedicle bone during the initial insertion and leaving a retention length to allow the screw depth to be adjusted.

## Figures and Tables

**Table 1:** Thread dimensions for the three pedicle screw designs.

Screw Design	OD (mm)	ID (mm)	Pitch (mm)	Flank Angle (°)
SLT	6.0	4.4	2.5	10
DLT				20
MSLT			1.55/3.1 proximal/distal	10

**Table 2:** Pullout strength of screws without preconditioned cyclic loading.

Type of Screw	Average Pullout Strength (N) under Different Insertion Strategy		
	A	B	C
SLT (group 1)	1709 ± 9	1509 ± 16	1528 ± 20
DLT (group 2)	1700 ± 17	1478 ± 19	1503 ± 21
MSLT (group 3)	1912 ± 15	1646 ± 19	1723 ± 12

**Table 3:** Pullout strength of screws with preconditioned cyclic loading.

Type of Screw	Average Pullout Strength (N) under Different Insertion Strategy		
	A	B	C
SLT (group 4)	1531 ± 20	1295 ± 23	1332 ± 12
DLT (group 5)	1443 ± 18	1137 ± 25	1250 ± 16
MSLT (group 6)	1722 ± 16	1098 ± 27	1386 ± 20

## Data Availability

All relevant data are within the manuscript.

## References

[1] Bokov A., Bulkin A., Aleynik A., Kutlaeva M., Mlyavykh S. (2019). Pedicle Screws Loosening in Patients With Degenerative Diseases of the Lumbar Spine: Potential Risk Factors and Relative Contribution. Glob. Spine J..

[2] Galbusera F., Volkheimer D., Reitmaier S., Berger-Roscher N., Kienle A., Wilke H.-J. (2015). Pedicle screw loosening: A clinically relevant complication?. Eur. Spine J..

[3] Bokov A., Pavlova S., Bulkin A., Aleynik A., Mlyavykh S. (2021). Potential contribution of pedicle screw design to loosening rate in patients with degenerative diseases of the lumbar spine: An observational study. World J. Orthop..

[4] Peck J.H., Cadel E., Palepu V., Ferrell B.M., Warner C.H. (2021). Mechanical performance of thoracolumbosacral pedicle screw systems: An analysis of data submitted to the Food and Drug Administration. J. Biomech..

[5] Warburton A., Girdler S.J., Mikhail C.M., Ahn A., Cho S.K. (2020). Biomaterials in Spinal Implants: A Review. Neurospine.

[6] Hart R., Hermsmeyer J.T., Sethi R.K., Norvell D.C. (2015). Quality and Quantity of Published Studies Evaluating Lumbar Fusion during the past 10 Years: A Systematic Review. Glob. Spine J..

[7] Korovessis P., Papazisis Z., Koureas G., Lambiris E. (2004). Rigid, Semirigid Versus Dynamic Instrumentation for Degenerative Lumbar Spinal Stenosis: A Correlative Radiological and Clinical Analysis of Short-Term Results. Spine.

[8] Weegens R., Carreon L.Y., Voor M., Gum J.L., Laratta J.L., Glassman S.D. (2022). Dual pitch screw design provides equivalent fixation to upsized screw diameter in revision pedicle screw instrumentation: A cadaveric biomechanical study. Spine J..

[9] Seng W.R.D., Chou S.M., Siddiqui S.S., Oh J.Y. (2019). Pedicle Screw Designs in Spinal Surgery: Is There a Difference? A Biomechanical Study on Primary and Revision Pull-Out Strength. Spine.

[10] Çetin A., Bircan D.A. (2021). Experimental investigation of pull-out performance of pedicle screws at different polyurethane (PU) foam densities. Proc. Inst. Mech. Eng. Part H J. Eng. Med..

[11] Ying S.-H., Kao H.-C., Chang M.-C., Yu W.-K., Wang S.-T., Liu C.-L. (2012). Fixation Strength of PMMA-augmented Pedicle Screws After Depth Adjustment in a Synthetic Bone Model of Osteoporosis. Orthopedics.

[12] Matityahu A., Hurschler C., Badenhop M., Stukenborg-Colsman C., Waizy H., Wentz B., Marmor M., Krettek C. (2013). Reduction of Pullout Strength Caused by Reinsertion of 3.5-mm Cortical Screws. J. Orthop. Trauma.

[13] Wu L.-C., Hsieh Y.-Y., Tsuang F.-Y., Kuo Y.-J., Chiang C.-J., Cheng C.-K., Tsai T.-Y., Wang L., Ai S., Wu L.-C. (2022). Cutting Flute and Thread Design on Self-Tapping Pedicle Screws Influence the Insertion Torque and Pullout Strength. Appl. Sci..

[14] Chao K.-H., Lai Y.-S., Chen W.-C., Chang C.-M., McClean C.J., Fan C.-Y., Lin L.-C., Cheng C.-K. (2013). Biomechanical analysis of different types of pedicle screw augmentation: A cadaveric and synthetic bone sample study of instrumented vertebral specimens. Med. Eng. Phys..

[15] Mehmanparast H.N., Mac-Thiong J.M., Petit Y. (2012). Compressive Properties of a Synthetic Bone Substitute for Vertebral Cancellous Bone. Int. J. Biomed. Biol. Eng..

[16] Nagaraja S., Palepu V. (2016). Comparisons of Anterior Plate Screw Pullout Strength Between Polyurethane Foams and Thoracolumbar Cadaveric Vertebrae. J. Biomech. Eng..

[17] Standard Specification for Rigid Polyurethane Foam for Use as a Standard Material for Testing Orthopaedic Devices and Instruments. https://www.astm.org/f1839-08r21.html.

[18] Standard Specification and Test Methods for Metallic Medical Bone Screws. https://www.astm.org/f0543-07.html.

[19] Burval D.J., McLain R.F., Milks R., Inceoglu S. (2007). Primary Pedicle Screw Augmentation in Osteoporotic Lumbar Vertebrae: Biomechanical Analysis of Pedicle Fixation Strength. Spine.

[20] Wadhwa R.K., Thakur J.D., Khan I.S., James J., Ahmed O., Zhang S., Henderson B., Ogden A., Guthikonda B., Nanda A. (2015). Adjustment of Suboptimally Placed Lumbar Pedicle Screws Decreases Pullout Strength and Alters Biomechanics of the Construct: A Pilot Cadaveric Study. World Neurosurg..

[21] Karami K.J.D., Buckenmeyer L.E.M., Kiapour A.M., Kelkar P.S.D., Goel V.K., Demetropoulos C.K., Soo T.M. (2015). Biomechanical Evaluation of the Pedicle Screw Insertion Depth Effect on Screw Stability Under Cyclic Loading and Subsequent Pullout. J. Spinal Disord. Tech..

